# Supplementary material for: Functional Characterization of the CpNAC1 Promoter and Gene from Chimonanthus praecox in Arabidopsis
Source: Int J Mol Sci. 2022 Dec 29;24(1):542. doi: 10.3390/ijms24010542 (PMC9820485; doi:10.3390/ijms24010542)
Supplement: Supplementary file 1 [file ijms-24-00542-s001.zip › ijms-2089746-supplementary.pdf]

Table S1. List of primers

Primer name	Primer sequences (5'-3')
CpSNAC1-F	GATCAAGTGACCTTAGCCCATC
CpSNAC1-R	AGGAGATGAGATGATACTGAGGC
CpSNAC1-ORF-F	tccccccggggGAACAGCAATCGGTTGTGAGGC
CpSNAC1-ORF-R	gcgtcgacGGTGAGTTGTAGCCTGGGTATG
VP16-F	catgccatggGAACAGCAATCGGTTGTGAGGC
VP16-R	GCAGTGGCAGACTCACATAGGGTGAGTTGTAGCCTGGGTATG
CpSNAC1-VP16-F	GAACAGCAATCGGTTGTGAGGC
CpSNAC1-VP16-R	gcgtcgacGGTGAGTTGTAGCCTGGGTATG
pCAMBIA1300-*GFP-CpSNAC1-F*	gcgtcgcgacTCTAAAACCAGGAGGCAG
pCAMBIA1300-*GFP-CpSNAC1-R*	cgggatccGTCATGGGTTGATGGGCA
qCpSNAC1-F	AAGCCTGAACTTCTTACAAATGCC
qCpSNAC1-R	ACTTTGGCTGGCTCTGGACC
qActin-F	GTTATGGTTGGGATGGGACAGAAAG
qActin-R	GGGCTTCAGTAAGGAAACAGGA
qTublin-F	TAGTGACAAGACAGTAGGTGGAGGT
qTublin-R	GTAGGTTCCAGTCCTCACTTCATC
CpSNAC1-SP1	GCTCTTCCTCCATCTCATTCTCCCTGC
CpSNAC1-SP2	GAACAGCAATCGGTTGTGAGGCG
CpSNAC1pro-pst1-F	AACTGCAGGAGAGTTGGATGATGAGAAGAAGAT
CpSNAC1pro-nco1-R	CATGCCATGGGAACAGCAATCGGTTGTGAGGC
CpSNAC1-D1-pst1-F	AACTGCAGCAAGTAGGGCTAAATAACCCATTTACC
CpSNAC1-D2-pst1-F	AACTGCAGATGTTGTTGCACGAGCGATCAAT
CpSNAC1-D3-pst1-F	AACTGCAGGTTTTCCAACACGAGCCCTCTCT
CpSNAC1-nco1-R	CATGCCATGGGAACAGCAATCGGTTGTGAGGC
GUS-F	CATCCTCTGGGAACCACTGAAC
GUS-R	CATCACATTGCTCGCTTCGTT
pCAMBIA1300- *CpSNAC1-F*	gcgagctcAACAGCAATCGGTTGTGAGGC
pCAMBIA1300- *CpSNAC1-R*	gctctagaACTTTGGCTGGCTCTGGACCT

Table S2. ***cis*****-regulatory elements in the** ***CpSNAC1*** **promoter**

Factor or cis-regulatory element	No.of cis-regulatorys	Core sequence	Description
ABRE	11	TACGTG/ACGTG/ CGCACGTGTC /GACACGTGGC/ CACGTG	cis-acting element involved in the abscisic acid
ARE	2	AAACCA	cis-acting regulatory element essential for the anaerobic induction
AuxRR-core	1	GGTCCAT	cis-acting regulatory element involved in auxin responsiveness
Box 4	4	ATTAAT	part of a conserved DNA module involved in light responsiveness
CAT-box	2	GCCACT	cis-acting regulatory element related to meristem expression
CAAT-box	36	CCAAT/ CAAT/ CAAAT/ CAAAT	common cis-acting element in promoter and enhancer regions
G-Box	3	CACGTG	cis-acting regulatory element involved in light responsiveness
G-box	9	TACGTG/CACGTG/ ACACGTGT/ CACGTGG	cis-acting regulatory element involved in light responsiveness
CGTCA-motif	3	CGTCA	cis-acting regulatory element involved in the MeJA-responsiveness
GARE-motif	2	TCTGTTG	gibberellin-responsive element
MBS	1	CAACTG	MYB binding site involved in drought-inducibility
MYB	2	CAACAG	responds to dehydration and ABA signals
MYC	4	CATTTG	responds to abiotic stress signals
P-box	1	CCTTTTG	gibberellin-responsive element
Sp1	1	GGGCGG	light responsive element
TATA-box	28	ATTATA/TATAA/TATA/ TACAAAA/TAAAGATT/ TATACA	core promoter element around -30 of transcription start
TGACG-motif	3	TGACG	cis-acting regulatory element involved in the MeJA-responsiveness
